# Is platelet activating factor (PAF) an important mediator in bronchial asthma?

**DOI:** 10.1155/S0962935192000541

**Published:** 1992

**Authors:** R. H. Gundel, H. O. Heuer, L. G. Letts

**Affiliations:** Department of Pharmacology Boehringer Ingelheim Pharmaceuticals Inc. Ridgefield CT 06877 USA

## Abstract

The development of selective PAF receptor antagonists may provide a novel approach to the treatment of human bronchial asthma. In preclinical animal models of human asthma, PAF receptor antagonists have been found to be efficacious in blocking antigen-induced changes in lung function. However, the majority of these models involve acute inflammatory events and transient changes in lung function and, therefore, their relevance to human asthma is questionable. In a recent study with a primate model of chronic airway inflammation and hyperresponsiveness, we have shown that treatment with a PAF receptor antagonist had no effect on reducing chronic inflammation and hyperresponsiveness. Similarly, recent studies in human asthmatics with PAF receptor antagonists have failed to show efficacy in blocking allergen-induced airway responses or to have any steroid sparing effects in patients with ongoing asthma. Thus, it seems that PAF may not be a key mediator which can be blocked and thereby provide therapy for bronchial asthma.


Mediators of Inflammation 1, 367–369 (1992)

### Is platelet activating factor (PAF) an important mediator in bronchial asthma?

R. H. Gundel,[CA] H. O. Heuer and L. G. Letts

Department of Pharmacology, Boehringer Ingelheim Pharmaceuticals, Inc., Ridgefield, CT 06877, USA

[CA] Corresponding Author

**THE development of selective PAF receptor antagonists may provide a novel approach to the treatment of human bronchial asthma. In preclinical animal models of human asthma, PAF receptor antagonists have been found to be efficacious in blocking antigen-induced changes in lung function. However, the majority of these models involve acute inflammatory events and transient changes in lung function and, therefore, their relevance to human asthma is questionable. In a recent study with a primate model of chronic airway inflammation and hyperresponsiveness, we have shown that treatment with a PAF receptor antagonist had no effect on reducing chronic inflammation and hyperresponsiveness. Similarly, recent studies in human asthmatics with PAF receptor antagonists have failed to show efficacy in blocking allergen-induced airway responses or to have any steroid sparing effects in patients with ongoing asthma. Thus, it seems that PAF may not be a key mediator which can be blocked and thereby provide therapy for bronchial asthma.**

**Key words:** PAF, Asthma, PAF receptor antagonists.

Bronchial asthma is a disease that affects approximately 5–8% of the population and despite the availability of more potent, selective therapies, the incidence of asthma morbidity and mortality is increasing. Asthma is a complex disease that is characterized, in part, by a reversible, episodic bronchoconstriction and wheeze. The airways of asthmatics are inflamed and hyperresponsive making them respond (constrict) to a variety of stimuli.

Over the past few years it has become increasingly apparent that asthma has a strong inflammatory component. This realization has prompted an intense effort within academia and industry to define the role of inflammatory cells and mediators in asthma. The hypothesis that PAF is an important mediator in the pathophysiology of asthma was initially derived from studies demonstrating its ability to constrict airway smooth muscle at very low doses[1] as well as its pro-inflammatory properties.[2] While the broncho-constrictive and oedema-inducing effects of PAF are shared by other putative mediators of inflammation such as histamine, thromboxane and leukotrienes, PAF is the only mediator which simultaneously increases mucus secretion[3] and recruits platelets and eosinophils[4] from the extravascular space into the lungs. Since eosinophils are thought to play an important role in the pathogenesis of asthma, it is of interest therefore, that PAF is among the most potent chemotactic factors for eosinophils.[5] Furthermore, similar to antigen inhalation, inhaled PAF induces an acute and late-phase response in dual responder animals[6] and, in addition, has been shown to induce bronchial hyperreactivity in different species including man.[7] Thus, in light of the biology of PAF, the development of selective PAF-receptor antagonists seemed most promising in terms of a novel approach to the treatment of bronchial asthma.

*PAF antagonists in preclinical animal models:* Studies of PAF antagonists in preclinical models of asthma have produced encouraging results demonstrating potent inhibition of allergen induced bronchoconstriction, infiltration of eosinophils into the airways, lung oedema formation, late phase reactions and the development of nonspecific bronchial hyperresponsiveness. In addition, the formation and release of inflammatory mediators (including leukotrienes, prostaglandins, thromboxanes and histamine) have been shown to be inhibited by selective PAF receptor antagonists *in vivo*, although a direct interaction of the PAF antagonists with these mediators has been excluded. The role of PAF in a sequence of pathophysiological events involving other mediators, and the possibility of interactive synergism, strengthens the view that antagonists of PAF may have effects extending beyond those assumed for a single mediator. In models of *in vivo* anaphylaxis the divergent results obtained with PAF antagonists may be due to differing experimental conditions (e.g. testing in the absence of a small dose of an antihistamine, antigen load during challenge, booster sensitization etc.) and the different potency and bioavailability of PAF

© 1992 Rapid Communications of Oxford Ltd antagonists *in vivo*. PAF antagonists seem to work more consistently in passive anaphylaxis and during challenge with the antigen by the inhaled route.[8] In active anaphylaxis PAF antagonists are more effective when animals are once sensitized compared to a booster sensitization regimen,[9,10] however, PAF antagonists can still block anaphylaxis in boosted animals.[10] With respect to microvascular permeability, in one set of experiments a PAF antagonist failed to block leakage at a dose which inhibited PAF induced microvascular leakage.[11] Other results are in favour of a PAF antagonist blocking antigen induced microvascular leakage.[12]

PAF antagonists block eosinophil and leukocyte infiltration in the bronchial tissue and appearance of the cells in bronchoalveolar fluid of sensitized guinea pigs,[4] rabbits[13] and monkeys.[14] A few studies have, however, failed to show this effect. Inhibition of the late phase response to antigen with selective PAF antagonists has been demonstrated in several animal models including allergic guinea-pigs,[15] rabbits,[16] sheep[6] and monkeys.[14] Inhibition of antigen increased bronchial responsiveness by selective PAF antagonists has been reported by several groups in guinea-pigs,[11] rabbits[13] and other species.[17] The experimental conditions in the studies cited above involve a single antigen challenge to sensitized animals with normal (uninflamed) lungs and the relevance of these preclinical models to human bronchial asthma remains unclear.

*PAF antagonists in clinical studies:* In contrast to supportive results from preclinical models of asthma, the clinical data obtained so far are not in favour of a major role of PAF in human bronchial asthma.[18–20] Despite the fact that several clinical studies have documented that PAF antagonists block exogenous PAF induced effects in man,[21,22] oral or inhaled PAF antagonists have failed to block antigen induced early or late phase responses or increases in bronchial responsiveness. In addition, there is also no support for clinical efficacy from a recent phase II steroid sparing study in ongoing human bronchial asthma (S. Holgate, personal communication). One may speculate on the possible reasons for the discrepancy between supportive preclinical results and failure of PAF antagonists in clinical human asthma. The first and most obvious conclusion that can be drawn from the clinical data is that, in contrast to different animal species (including monkeys), PAF is not an important mediator in human asthma. Secondly, although there is no convincing evidence that different subtypes of PAF receptors exist in lung tissue and inflammatory cells present in lung tissue, there may be subtypes of PAF receptors including intracellular receptors with varying affinities for PAF and PAF antagonists. This question deserves further investigation. Third, due to priming and synergism of PAF with other mediators of inflammation produced during chronic asthma, the doses of PAF antagonists needed may be far higher than those needed to antagonize exogenous PAF.[23] However, it is also conceivable that a high dose therapy of PAF antagonists may result in an up-regulation of PAF receptors or expression of other ligands involved in the inflammatory process. This latter point emphasizes the importance of the dosing regimen and the possibility of a rebound phenomenon. Finally, the question remains open whether only subtypes of asthma (severe, mild, intrinsic, extrinsic) are sensitive to PAF antagonists and whether the appropriate clinical target parameter has been addressed in recent clinical studies. This complexity of disease diagnosis further illustrates and exposes the limitations of preclinical models.

In conclusion, based on available clinical studies so far, it seems that platelet activating factor is not a key mediator which can be blocked and thereby provide therapy in human bronchial asthma. However, several critical questions of its physiological function remain unanswered. In preclinical settings PAF antagonists have only been shown to be effective in single challenge, acute disease related models when the action of other mediators could be inhibited indirectly via their release being subsequent to a synergism with PAF. These data could be interpreted that an obvious limitation of all preclinical models is the lack of a baseline chronic airway inflammatory setting. It may well reflect that there is a basic, underlying abnormality in lung function which influences, in part, the release and actions of inflammatory mediators. It needs to be demonstrated that in humans, the antagonism of PAF goes beyond the antagonism of this single mediator to have implications for any disease including bronchial asthma. It seems unlikely that the transient release of a single mediator or transient cell infiltrate may account for the complex pathophysiology of bronchial asthma. Bronchial asthma is a chronic disease and the results of a study from our laboratory with a PAF antagonist reported in this issue illustrates the possible limitations of preclinical models and the potential dangers of too little information.

### References

1. Vargaftig BB, Lefort J, Chignard M, Benveniste J. Platelet-activating factor induces a platelet-dependent bronchoconstriction unrelated to the formation of prostaglandin derivatives. *Eur J Pharmacol* 1980; **65:** 185–192.
2. Camussi G, Pawlowski J, Tetta C. Acute lung inflammation induced in the rabbit by local instillation of 1-O-octadecyl-2-acetyl-sn-glyceryl-3-phosphorylcholine or of native platelet activating factor. *Am J Pathol* 1983; **112:** 78–84.
3. Hahn HL, Purnama I, Lang M, Sonnwald U. Effects of platelet activating factor on tracheal mucus secretion, on airway mechanics and on circulating blood cells in live ferrets. *Eur J Resp Dis* 1986; **146:** 277–283.
4. Lellouch-Tubiana A, Lefort J, Simon MT, Pfister A, Vargaftig BB.

Eosinophil recruitment into guinea-pig lungs after PAF-acether and allergen administration: modulation by prostacyclin, platelet depletion and selective antagonists. *Am Rev Resp Dis* 1988; **137**: 948–954.

5. Wardlaw AJ, Moqbel R, Cromwell O, Kay AB. Platelet activating factor. A potent chemotactic and chemokinetic factor for human eosinophils. *J Clin Invest* 1986; **78**: 1701–1706.
6. Stevenson JS, Tallent M, Blinder L, Abraham WM. Modification of antigen-induced late responses with an antagonist of platelet activating factor (WEB 2086). *Fed Proc* 1987; 1461.
7. Cuss FM, Dixon CMS, Barnes PS. Effects of inhaled platelet activating factor on pulmonary function and bronchial responsiveness in man. *Lancet* 1986; **2**: 189–192.
8. Heuer H, Casals-Stenzel J. Effect of the PAF-antagonist WEB 2086 on anaphylactic lung reaction: Comparison of inhalative and intravenous challenge. *Agents and Actions* 1988; **Suppl.23**: 207–210.
9. Desquand S, Lefort J, Dumarey C, Vargaftig BB. The booster injection of antigen during active sensitization of guinea pigs modifies the anti-anaphylactic activity of the Paf-antagonist WEB 2086. *Br J Pharmacol* 1990; **100**: 217–222.
10. Heuer HO. WEB 2347: Pharmacology of a novel very potent and long acting hetrazepinoic paf-antagonist and its action in repeatedly sensitized guinea pigs. *J Lipid Mediators* 1991; **4**: 39–44.
11. Evans TW, Dent G, Rogers DF, Aursudkij B, Chung KF, Barnes PJ. Effect of a PAF antagonist, WEB 2086, on microvascular leakage in the guinea-pig and platelet aggregation in man. *Br J Pharmacol* 1988; **94**: 164–168.
12. Christy LJ, Stewart AG, Dusting GJ. Platelet-activating factor and leukotrienes in rat pulmonary anaphylaxis. *Proc Australian Physiol Pharmacol Society* 1988; **19**: 94.
13. Metzger WJ, Ogden-Ogle C, Atkinson LB. Chronic platelet activating factor (paf) aerosol challenge induces *in vivo* and *in vitro* bronchial hyperresponsiveness. *FASEB J* 1988; **2**: A1252.
14. Gundel RH, Tocellini CA, Clarke CC, Desai SN, Letts LG. The PAF receptor antagonist WEB 2170 inhibits antigen-induced mediator release and late-phase bronchoconstriction in primates. *J Allergy Clin Immunol* 1991; **87**: 308.
15. Hutson PA, Holgate ST, Church MK. Effect of WEB 2086 on early and late airway responses to ovalbumin challenge in conscious guinea-pigs. *Br J Pharmacol* 1988; **95**: 770P.
16. Coyle AJ, Urwin SC, Page CP, Touvay C, Villain B, Braquet P. The effect of the selective PAF-antagonist BN 52021 on PAF and antigen induced bronchial hyperreactivity and eosinophil accumulation. *Eur J Pharmacol* 1988; **148**: 51–58.
17. Soler M, Sielczak MW, Abraham WM. Platelet-activating factor (PAF) contributes to antigen-induced airway hyperresponsiveness and inflammation in allergic sheep: modulation by a selective PAF-antagonist. *J Appl Physiol* 1989; **67**: 406–413.
18. Bel EH, De Smet M, *et al.* The effect of a specific oral PAF-antagonist, MK-287, on antigen-induced early and late asthmatic reactions in man. *Am Rev Resp Dis* 1991; **143**: A811.
19. Freitag A, Watson RM, *et al.* The effect of treatment with an oral platelet activating factor antagonist (WEB 2086) on allergen induced asthmatic responses in human subjects. *Am Rev Resp Dis* 1991; **143**: A157.
20. Wilkens H, Wilkens JH, *et al.* Effects of inhaled PAF-antagonists (WEB 2086) on allergen-induced early and late asthmatic responses and increased bronchial responsiveness to methacholine. *Am Rev Respir Dis* 1991; **143**: A812.
21. Adamus WS, Heuer H, Meade CJ, Brecht HM. Safety, tolerability and pharmacologic activity of multiple doses of the new platelet activating factor antagonist WEB 2086 in human subjects. *Clin Pharmacol Ther* 1988; **45**: 270–276.
22. Heuer H, Adamus WS. Safety and pharmacological activity of oral bepafant (WEB 2170) in human volunteers. *J Lipid Mediators* 1990; **2**: 202–207.
23. Heuer H. Effect of the hetrazepinoic platelet-activating factor antagonist bepafant (WEB 2170) in models of active and passive anaphylaxis in mice and guinea pigs. *Lipids* 1991; **26**: 1374–1380.

**Received 14 August 1992;
accepted 14 September 1992**
